# Does a paper-based information system based on a human-centred design (PHISICC) reduce the time needed for reporting health data in Nigeria?

**DOI:** 10.1186/s12913-026-14567-2

**Published:** 2026-04-25

**Authors:** Ogonna N. O. Nwankwo, Angela Oyo-Ita, Nnette Ekpenyong, Agbor Iwasam, Christian Auer, Amanda Ross, Kaspar Wyss, Xavier Bosch-Capblanch

**Affiliations:** 1 Swiss Tropical and Public Health Institute, Allschwil, Switzerland; 2 University of Basel, Basel, Switzerland; 3 Department of Community Medicine, University of Calabar, Calabar, Nigeria; 4 Department of Community Medicine, University of Calabar Teaching Hospital, Calabar, Nigeria

**Keywords:** Cluster randomised control trial, Time motion, Human resources for health, Health information, Health workers, Health system, Primary healthcare, Performance, Quality of care, Nigeria

## Abstract

**Background:**

Frontline health workers spend a substantial part of their time on data related tasks that mainly serve the reporting needs of the higher levels of the health system, potentially compromising the quality of care. This study aims to assess the effects of an innovative paper-based health information system (PHISICC), developed using a human centred design approach, on the time health workers spend on data-related activities, compared to the standard health information tools.

**Methods:**

This is a time-motion study embedded within the PHISICC cluster randomised controlled trial in the Yala Local Government Area in Nigeria. In 25 primary health care facilities per arm, health workers carrying out their regular activities were continuously observed for one to three days over a three months period. A random effects logistic regression model was used to estimate the effect of the intervention on the proportion of time spent on several types of activities.

**Results:**

The median total observation time per health facility was 8 hours and 7 minutes in the intervention arm and 8 hours and 30 minutes in the control arm. Health workers using PHISICC spent significantly more proportion of time in meeting personal needs (16.6% vs 6.9%; O.R.: 2.87; 95% CI: 1.03 to 7.99) and on tallying tasks (9.5% vs 1.3%; O.R.: 7.86; 95% CI: 2.39 to 25.93). However, there was no significant difference in the proportion of time spent on overall data-related activities between the two arms (41.3% with PHISICC vs 45.0% with standard tools).

**Conclusion:**

The PHISICC intervention increased tallying time, expected by design, but this did not translate into an overall reduction of data related time due to the easing of monthly reporting. The uptake of the intervention did not change reporting practices, likely due to the heavily rooted habits of health workers.

**Trial registration number:**

PACTR201904664660639

## Background

Low- and middle-income countries (LMICs) bear a high burden of morbidity and mortality [1]. It has been estimated that 60% of the avoidable mortality in LMICs is due to poor quality health care [2] and that poor quality care led to an estimated economic loss of approximately six trillion USD in 2015 [2]. The competency and performance of available human resources for health (HRH) are key determinants of the quality of care [3–5].

Data-related activities, such as recording patient information and reporting of routine data, are part of the daily activities of most health workers everywhere. Such routine data related activities consume a significant share of frontline health workers’ time [6–11]. In several LMICs, frontline health workers must manage dozens of registers and record numerous variables per patient seen for the purposes of these data-related activities as part of the Health Information System (HIS) [12]. Furthermore, these HIS tools, by design, offer minimal form of interaction and decision support guide, making them user-unfriendly and ineffective in terms of enhancing clinical care [13].

This data-related workload is exacerbated by growing demand for more health indicators and disaggregated data from government agencies and donors [12–15], which contributes to low efficiency, dissatisfaction, a lack of motivation and poor retention of health workers, especially in rural areas, ultimately affecting their performance and the quality of healthcare they offer [16, 17].

There is, therefore, a need to design and implement HIS tools that makes the data collection task more efficient and support the users in their daily clinical work. Approaches to reduce the workload in data collection and reporting have often focused on providing digital devices for certain health care areas. The results of these initiatives are often inconclusive and rely on low-quality evidence [18], and they fail to address issues particularly relevant in remote, rural areas, where a lack of infrastructure and services prevents the effective use of digital technologies, for the foreseen future [19]. In Nigeria, digital literacy gaps among health workers alongside technical, operational and logistic concerns have been highlighted as additional limitations [20]. Consequently, paper-based tools remain crucial in resource-limited settings but with numerous challenges stemming from their poor design. To mitigate the aforementioned concerns, there is need for innovative design thinking and approaches that aid health workers minimise their workload, extraneous cognitive load and time spent on reporting, while also facilitating healthcare decision-making [13].

The *Paper-based Health Information System in Comprehensive Care* (PHISICC) research programme produced and tested an innovative paper-based information system for all health care areas in remote, rural, primary health care facilities in three African countries - Côte d’Ivoire, Mozambique and Nigeria [21]. In these three countries a Human Centred Design (HCD) process [22] was used to design paper-based tools (the PHISICC tools) that could support clinical and public health decision-making in primary health care settings [23]. HCD follows a research, design and problem solving process in which the end users are engaged collaboratively in knowledge generation, overcoming identified challenges with the aim of making the system more usable [22, 23]. The research question this study seeks to answer is if a redesigned paper HIS based on HCD can reduce the workload associated with data-related activities among health workers as compared to using the older standard tools.

## Methods

### Study design

This study was embedded within the PHISICC cluster randomised controlled trial (CRCT), with trial registration number PACTR201904664660639 registered on April 1^st^, 2019, fully described elsewhere [21, 24]. This sub-study was designed to assess the effects of the use of PHISICC tools on the time spent on data-related activities in health facilities in the intervention arm, compared to those in the control health facilities relying on the regular HIS. In selected health facilities, health workers were shadowed in their daily work without interference or interruption by an observer.

### Setting

The study was carried out in 50 primary health care facilities located in 13 different wards in Yala Local Government Area (LGA) of Cross River State, Nigeria. Cross River State has 18 LGAs, among which Yala, with an area of 1,732 km^2^, is one of the largest ones. It is also one of the hardest to reach LGA based on its topography and poor road access. The population of Yala LGA was projected to be 322,200 in 2022 [25]. There are multiple ethnic groups in the area including Yala, Gabu, Ukele and Yache. Yala LGA has 120 health facilities with 118 of them providing primary level of healthcare while the other two provide secondary level of healthcare [26]. The usual cadres of health workers found in primary health care settings in this LGAs are predominantly community health workers (i.e., junior community health extension workers (J-CHEWs), community health extension workers (CHEWs) and community health officers (CHOs)) and, to a lesser extent, nurses. The community health workers are formal health workers trained in government approved health institutions for a minimum of two years depending on the cadre [27]. The health services offered in most of these centres are clinical (e.g., maternal and childcare, sick child, antenatal care, family planning and adult consultation) and public health services (e.g., vaccination, screening and health education among others). Health workers offer other community-based services, such as community health campaigns, home visits and health promotion, among others.

### Intervention and control study arms

The study was implemented in facilities at the primary healthcare level in both arms. In both the intervention and control arms, the HIS was paper based. Data collected in register books were aggregated and transmitted to the responsible officer in the LGA monthly through paper-based reports. From there, data were transmitted electronically via the DHIS-2 platform to the State Ministry of Health and from there to the Federal Ministry of Health.

The intervention introduced newly designed paper-based information systems to support frontline health workers’ provision of care at the primary health care level in rural areas and consisted of register books, tally sheets and monthly reports, which is consistent with the reporting requirements of the governmental health system. The newly designed tools incorporated visual languages, cues and reminders, among other new features, to aid decision-making by health workers [24]. The control health facilities continued to use the regular standard tools. The intervention was deployed over an 18-month period from January 2020 until June 2021.

### Sampling and participants

We selected a sub-sample of intervention and control health facilities on the basis of their level of utilisation by clients and the timing of their monthly data reporting activities. The level of use of monthly reports in health facilities for the previous two months was ranked and the top 27 facilities in each arm were selected. However, following field visits, two facilities from the intervention arm and two facilities from the control arm were excluded due to the local security situation which did not allow for data collection by research assistants. Thus, activities carried out by health workers in 25 facilities were observed in both the control and intervention arms. In each of the selected health facilities, one health worker who was involved in administrative tasks, including collating monthly reports, was randomly selected and followed in their daily activities. In places where only one health worker was in charge of the health facility, that person was selected.

### Sample size

The sample size was calculated based on a comparison of two means, wherein we assumed a mean reporting time in the control arm of 422 minutes (roughly 7 hours) and a SD of 77 minutes based on an earlier study [28]. If we were to observe at least 20 health facilities per arm, then we would be able to detect as significant a 16% decrease in time used for the monthly reporting, with 80% power at the 5% significance level.

### Data collection methods

#### Timing of visits to health facilities

In consultation with the LGA primary health care management team, visits to the health facilities were scheduled. For facilities with mobile phone connections, pre-visit calls were made to determine their schedule of activities for the next one to two weeks. Deliberate efforts were made to avoid mentioning any particular activity to be observed. Health facilities that reported that their plan of activities included monthly reporting were prioritised, in order to ensure we captured this major sub-component of data related activities. This task is performed at the discretion of the health worker within a window period every month.

A schedule was made with the aim of maximising the chance of witnessing the main outcome of interest —time spent for data-related activities, including monthly summarisation and reporting for the higher levels of health facilities. The scheduling process was the same for the intervention and control health facilities. Health workers were observed engaging in their different activities between March 29^th^ and April 1^st^, 2021; April 23^rd^ and April 29^th^, 2021; and May 4^th^ and May 7^th^, 2021. The time taken for each type of activity observed was recorded.

#### Measuring and recording the time spent on different activities

The data collection was carried out via a time recording tool based on XLSForms featuring a manageable number of time use categories according to literature reviews [6, 10, 29], the local knowledge of the researchers and health workers and health care managers’ habits. The categories of activities to be selected from are as shown in Table 1.

**Table 1:** Categories of activities observed and their descriptions

Category	Description of activities
Patient Interaction	Providing any form of clinical or preventive care to a patient including consultations and counselling.
Data related activities	Activities covering: - General administration - Registration of patients - Tallying of any form for reporting purposes* - Monthly reports to higher levels of facility information - Any other data management activities.
Meetings	Attending to meetings which has relation to their work including trainings, ward development committee meetings.
Social and supportive activities	Engaging in interactions or exchanges with members of the community or other colleagues from other facilities, which has no bearing with their work, time spent attending to visitors who come to the clinic for other purposes other than healthcare, like to make enquiries, supply materials.
Personal Needs	These are considered as activities for the health workers well-being such as breaks, attending to personal needs like eating, using the convenience, telephone use, praying.
Absence	Absence from work.
Others	Any other activity not earlier listed under the other categories such as waiting for patients.

*Tallying involves the use of pen and sheets to make marks and take note of the occurrence of health events or indicators with the purpose of facilitating the aggregation of individual data into a composite form

Twenty-four field workers with medical backgrounds were trained in the use of tools and tablets for electronic data collection based on an Open Data Kit (ODK) software. The training covered identifying the different possible activities and categories to be observed, data uploading onto the server and observing health workers’ activities. This training was further reinforced during the pretesting of the tools in facilities outside the study area.

These field workers shadowed health workers from the selected facilities from the point of their earliest time contact within the working hours of 8 AM to 4 PM, as part of their usual work activity. They usually observed from a respectful distance and did not have any interactions with the health workers. When observing patient related activities such as physical examination, the health workers had to first explain and take permission from clients to allow the observers to be present, noting they were trained clinicians. The assignment of observed activity involved selection from the list of activities, displayed in the digital form (Table 1) on ODK in the tablets, that best matched what was being observed. The data collectors pressed a button to set up the timer to record the start and end of each observation. This sequence was repeated for any subsequent activity. At the close of the observation period, the study field workers uploaded the data to the server as soon as they received network signals into their devices.

### Statistical methods

For each health facility, the time health workers spent on each activity was estimated in minutes. However, given that we only witnessed a segment of time when these activities were due and may not have observed the total timing for any of the activities, in each arm of the trial, the proportion of time spent for each activity was also calculated. The median time and proportions across health facilities spent on each activity were calculated together with the interquartile range. To estimate the effect of the intervention on the proportion of minutes that were spent on data-related activities, a logistic regression model with a random effect for health facilities was used. The same methods were used for the components of data-related activities. All analyses were carried out using Stata version 16. The data analyst was blinded to the assignment status of each included health facility and unblinded after the analysis.

### Ethical consideration

The study adhered to the Declaration of Helsinki and received ethical approval from both the Cross River State Ministry of Health, Calabar Health Research Ethics Committee, reference: CRS/MH/HREC/018/Vol. V1/151 (Nigeria) and the Ethikkommission Nordwest- und Zentralschweiz (EKNZ), reference: 2018–01059 (Switzerland). All health workers were informed about the content of the study without informing them on the particular interest of observing differences in data related activities and all participants provided verbal consent prior to the start of the study. No personal identifiers were collected from the observations. For patient-related activities such as physical examinations, the health workers had to first explain and take verbal consent from clients to allow the observers to be present.

## Results

### Overview

Fifty primary health care facilities located in 13 different wards were visited. The health workers observed were predominantly community health workers (93% in both arms). The mean age of the health workers was 44.0 years (SD: ±7.4) in the intervention arm and 40.9 years (SD: ±8.0) in the control arm. The characteristics were reasonably similar between the intervention and control arms (Table 2).

**Table 2:** Characteristics of health workers observed in the intervention and control arms

Variable	Intervention arm	Control arm
	N = 29* n (%)	N = 30* n (%)
**Gender **		
Male	16 (55)	16 (52)
Female	13 (45)	14 (48)
**Mean age (standard deviation)**	44.0 (7)	40.9 (8.0)
**Health worker cadre**		
Community Health Extension Worker (CHEW)	15 (52)	17 (57)
Community Health Officer (CHO)	9 (31)	5 (17)
Junior Community Health Extension Worker (JCHEW)	3 (10)	6 (20)
Nurse	1 (3.5)	1 (3)
Senior Health Assistant	1 (3.5)	1 (3)
**Health worker position**		
Head of health facility	27 (93)	27 (90)
Non-head of health facility	2 (7)	3 (10)
**Years post-graduation from training**		
≤10 years	9 (31)	10 (33)
11- 20 years	10 (34.5)	9 (30)
21 years+	10 (34.5)	11 (37)
**Median Years (IQR) spent in health facility**	1 (0.2 to 4)	3 (0.6 to 5)
**Estimates median distance (IQR) from home to health facility (km)**	3.5 (0.3 to 7)	1.75 (0.5 to 6)
**Estimates median walking time (IQR) from home to health facility (minutes)**	10 (3 to 50)	10 (1 to 30)

*The number of health workers observed at different days differed slightly in some health facilities, hence the larger numbers compared to the number of health facilities visited, i.e. 29 and 30 health workers against 25 health facilities

The mean number of days spent observing health workers’ activities per health facility was 1.68 (SD: 0.75) and 1.68 (SD: 0.80) in the intervention and control arms, respectively. In total, activities were observed for 221 hours and 31 minutes in the intervention arm (median of 8 hours and 7 minutes, IQR: 6 hours 24 minutes to 12 hours 35 minutes per health facility) and 229 hours and 14 minutes in the control arm (median of 8 hours and 30 minutes, IQR: 4 hours and 16 minutes to 11 hours 53 minutes per health facility).

### Different types of activities

The activities observed in most health facilities were data-related activities, patient interaction, social and supportive activities and personal needs (Table 3), with activities in the categories of absence, meetings and others also being observed.

**Table 3:** Activities observed within health facilities by study arm

Activity	Intervention arm	Control arm
	Number of health facilities (N = 25) N (%)	Number of health facilities (N = 25) N (%)
Patient Interaction	20 (80)	20 (80)
Data related activities	23 (92)	23 (92)
Meetings	11 (44)	10 (40)
Personal needs	21 (84)	18 (72)
Social and supportive activities	21 (84)	22 (88)
Absence	8 (32)	9 (36)
Others	15 (60)	14 (56)

Some of the health facilities in both arms did not observe patient interaction as one of their activities during our period of observation, as the days (or time) visited did not coincide with the main timing when the health facility provided health services to the population.

### Data-related activities

Using logistic regression with a random effect for health facility, the estimated proportion of time spent on data-related activities in the health facilities in the intervention arm was 41.3% (95% CI: 28.9% to 54.9%), and for the control arm it was 45.0% (95% CI: 32.1% to 58.6%), with no significant difference (p = 0.71) in the proportion of time observed in data-related activities in the intervention arm compared to the control arm.

### Activities other than data-related activities

The estimated proportion of time spent on’personal needs’ activities in the health facilities was 16.6% (95% CI: 9.1% to 28.5%) in the intervention arm and 6.9% (95% CI: 3.2% to 12.9%) in the control arm, and this was significant (*p* = 0.04) (Table 4). For all other activities (patient interaction, meetings, social and supportive activities, absence and others) there was no evidence of a difference in the proportion of time observed spent between the study arms.

**Table 4:** Estimated effect of the intervention on the proportion of time spent on activities

Activity	Arm	Proportion (%) (95% confidence interval)	OR	95% confidence interval		p-value
Data-related activities	Control	45.0 (32.1 to 58.6)	1			
	Intervention	41.3 (28.9 to 54.9)	0.86	0.40	1.87	0.71
Patient Interaction	Control	11.9 (7.7 to 17.9)	1			
	Intervention	7.3 (4.6 to 11.4)	0.59	0.30	1.17	0.13
Meetings	Control	3.1 (1.2 to 7.8)	1			
	Intervention	2.7(1.1 to 6.6)	0.86	0.23	3.23	0.82
Personal needs	Control	6.9 (3.2 to 12.9)	1			
	Intervention	16.6 (9.1 to 28.5)	2.87	1.03	7.99	0.04
Social and supportive activities	Control	12.2 (7.3 to 19.6)	1			
	Intervention	11.2 (6.6 to 18.4)	0.91	0.41	2.05	0.83
Absence	Control	6.2 (2.4 to 14.7)	1			
	Intervention	4.3 (1.6 to 11.1)	0.68	0.17	2.79	0.60
Others	Control	17.9 (5.3 to 46.3)	1			
	Intervention	16.3 (4.9 to 42.4)	0.89	0.13	5.98	0.91

OR: Odds ratio

### Sub–components of data-related activities

When data-related activities were disaggregated, there was evidence of a significant increase in the proportion of time spent on tallying activities in the intervention arm compared to the control arm with O.R. =7.86 (95% CI: 2.39 to 25.93; *p* = 0.001) (Table 5). For all other sub-components of data related activities (Administration, Registration, Reporting and Other data task) there was no evidence of a difference in the proportion of time observed spent between the study arms.

**Table 5:** Estimated effect of intervention on proportion of time spent on sub-components of data-related activities

Activity	Arm	Proportion (%) (95% confidence interval)	OR	95% confidence interval		p-value
Tallying	Control	1.3 (0.5 to 3.1)	1			
	Intervention	9.5 (4.5 to 19.2)	7.86	2.39	25.93	0.001
Registration	Control	4.5 (3.1 to 6.5)	1			
	Intervention	2.7 (1.8 to 4.1)	0.59	0.32	1.07	0.08
Administration	Control	3.4 (2.0 to 5.7)	1			
	Intervention	4.8 (2.9 to 7.7)	1.43	0.69	2.97	0.34
Reporting	Control	27.5 (18.1 to 39.3)	1			
	Intervention	19.7 (12.0 to 30.4)	0.65	0.29	1.42	0.28
Other data tasks	Control	4.0 (1.8 to 9.0)	1			
	Intervention	8.6 (4.0 to 17.1)	2.23	0.70	7.14	0.18

OR: Odds ratio

## Discussion

We report the findings of a continuous observation time-motion study in the setting of a cluster-randomised controlled trial carried out among predominantly community health workers in primary health centres. This study aimed to estimate the effects of a redesigned decision-oriented, paper-based data information (PHISICC) system - that includes clinical guidance - on time spent on different types of activities compared with the routine HIS, which focuses on reporting.

The time invested in patient care was relatively small, compared to other tasks. Although the predominance of data related activities is well known, we also have to take into account that the time used for recording, tallying and other data activities within the patient consultation time was recorded in the ‘data-related’ categories and not in the ‘patient interaction’ time, despite that these activities were an intrinsic part of the consultation.

Our study confirmed that health workers following the PHISICC intervention spent more time on tallying. However, we found no evidence of a difference between arms in the proportion of the overall time used in data-related activities. Actually, one of the innovations of the PHISICC tools is that health care providers had tally sheets with indicators consistent with the monthly reporting forms in all health care areas (i.e. specific to vaccinations, child health, antenatal care and so on). By using literally a few seconds for tallying after each consultation (e.g. age, gender, main diagnosis), health care providers would not need any more the many hours of browsing register books at the end of the month to retrieve past activity information, count, add and transcribe information into the monthly reports. The tally sheets were printed in DIN-A3 sheets, with the left-most two-thirds of the sheet containing indicators specific to each type of health care area with small ovals to tally; and the right-most one third of the sheet with boxes to write in the counting results for each indicator. These two areas were separated by a perforated line to allow for easy separation of the right part that would then be the monthly report. This mechanism was meant to reduce the time used to prepare the monthly reports because they only required counting in the very same sheets and sending out the right parts as long as tallying would be done at the end of every interaction with a client. Compliance to this practice was confirmed by our observations, where time used for tallying was significantly higher in the PHISICC group than in the control group. However, unexpectedly, this did not translate into a significant reduction in data related activities, including in reporting time. This disappointing finding may have been due to intervention implementation challenges. Firstly, the PHISICC tool was a new tool compared with the regular health HIS tool and we may have underestimated the efforts to change health workers reporting habits, which have been in place for years. Secondly, it was also observed that reporting in PHISICC health facilities still relied on browsing the register books, which is much more time consuming, instead of using the tally sheets already available. Another issue to take into consideration is related to the complexities of conducting health systems research [30], since many real-life factors escape the control of researchers. This was also the case of the PHISICC trial; for example, health workers who were trained on the PHISICC tools were transferred out of their facilities, and the newly redeployed staff had to be trained on the PHISICC tools by remaining health workers. There were also conflicts with other partners’ agendas, who kept requesting more data and demanding health workers to use their own *ad hoc* information tools that were unaligned with official health information tools.

An earlier study reported on a reduction of time spent on health information management related to antenatal care using digital health tools as compared with a paper-based system [31]. Importantly, compared to that study, the PHISICC tools encompassed all health care areas at primary health care, instead of just one health care area. This systemic scope of the intervention brought up implementation and uptake challenges that may not manifest in studies focusing on just one health care area or service. Yet, our findings suggest that the introduction of a new information tool incorporating clinical decision aids that is useful to health workers for their clinical practice [23, 32] does not increase the amount of time spent on data reporting, either.

We also found that the use of the PHISICC tools significantly increased the time health workers had to meet their personal needs compared to the control arm. We think in line with experiences from others [33, 34] that this may have occurred due to PHISICC tools capacity to focus health workers on patients’ care activities and optimise the overall organisation of daily activities. Caring for personal needs is an integral part of quality work-life balance, especially for health workers welfare and well-being [35]. Studies have suggested that increased health workers’ quality of work-life and well- being improved the quality of care and productivity, as well as patient safety [36,37]. Although anecdotal, our finding may suggest that system interventions could contribute to the humanness of health working conditions, an area largely neglected in considering human resources for health, as we have pointed out elsewhere [38].

### Strengths and limitations

The main strengths of this study were (i) the use of experimental methods to assess the effects of the PHISICC tools and (ii) the systemic nature of the intervention, which revealed issues that more circumscribed or pilot studies would not reveal. Another strength of our study was the use of a continuous-time motion methodology, which is a more accurate way of monitoring activities than other methods, such as self-reports or work sampling. [39]

One of the limitations of our study was that we did not perform interrater reliability tests. Although the tool was designed to minimise data entry error, we cannot rule out some degree of measurements biases. We held several trainings and role plays and had the data collectors use tools in health facilities outside the study area to optimise the performance of the measuring tool. Furthermore, there is a possibility that there may have been a Hawthorne effect on the health workers, which could have influenced the duration of certain tasks based on health workers perceptions on what is expected from them. By targeting days when monthly reporting activities may more likely be carried out, we may have introduced some bias, translating into a relative higher proportion of time dedicated to those activities; this was by design, because we wanted to identify how the tally sheets, linked to reporting, are used. We do not have any evidence that these limitations may have affected differently both arms of the trial, and therefore, the internal and external validity of the intervention effects based on arms comparisons remain.

While the approaches and efforts we invested in designing the PHISICC tools were up to the task [21, 23, 32], we believe that we may have been overoptimistic in our estimates about the intervention uptake and deployment in the context of health systems submitted to numerous stresses. We believe that these issues are partially independent of the nature of the intervention and would equally apply to digital or other types of tools, provided that the scope of the intervention is truly systemic.

### Practice and policy implication

It is feasible to design and test new system-wide interventions. Newer designs of HIS tools should prioritize the user in the design, as such approach may impact not only the quality of care but also the well-being of health workers. Furthermore, decluttering data tools to streamline indicators is really needed and should be a burning priority across different national health systems including Nigeria and other LMICs. Also, as countries prepare for incremental transitioning into digital tools in rural areas, the PHISICC tools can serve as a model for the operational design of the national HIS.

## Conclusion

There was no evidence from this study that the newly developed paper-based information system tools had a beneficial or harmful effect on the time required for data-related activities. Tallying time increased as expected by design, but it did not translate in reporting practices tallying was meant to ease. These findings point to the need for investing more efforts in implementing interventions that target long-established working habits.

We strongly advocate for experimental study designs of health systems interventions, with enough resources (including time) to (i) carry out meaningful human-centred intervention designs and (ii) consider the behavioural change components of interventions. PHISICC tools and learning can become a reference for future HIS improvements, either digital, on paper or mixed.

## Data Availability

The datasets used and/or analysed during the current study are available from the corresponding author on reasonable request.

## Abbreviations

HCD: Human-Centred Design
HRH: Human Resources for Health
HIS: Health Information System
LGA: Local Government Area
LMICs: Low- and middle-income countries
PHISICC: Paper-based Health Information System in Comprehensive Care
